# Functions of NO-GC1 and NO-GC2 in pain processing

**DOI:** 10.1186/2050-6511-16-S1-A74

**Published:** 2015-09-02

**Authors:** Jonas Petersen, Evanthia Mergia, Oliver Drees, Ruirui Lu, Catherine Real, Andreas Friebe, Doris Koesling, Achim Schmidtko

**Affiliations:** 1 Institut für Pharmakologie und Toxikologie, Universität Witten/Herdecke, Witten, Germany; 2 Institut für Pharmakologie und Toxikologie, Ruhr-Universität, Bochum, Germany; 3 Physiologisches Institut, Universität Würzburg, Würzburg, Germany

## Background

Chronic pain in response to tissue inflammation (inflammatory pain) or nerve injury (neuropathic pain) is often unresponsive to currently available treatments. A large body of evidence indicates that production of nitric oxide (NO) and activation of NO-sensitive guanylyl cyclase (NO-GC) essentially contributes to the processing of chronic pain. NO-GC is a heterodimer consisting of one α subunit (α_1_ or α_2_) and one β_1_ subunit and exists in two isoforms (NO-GC1 and NO-GC2). However, the functional role of NO-GC1 and NO-GC2 in pain processing remains poorly understood. Here, we investigated the expression of NO-GC isoforms in pain-relevant tissues (dorsal root ganglia and the spinal cord) and characterized the nociceptive behavior of mice lacking α_1_ or α_2_ in models of acute nociceptive, inflammatory and neuropathic pain.

## Conclusion

Our behavioral data point to different and partly contrary functions of NO-GC1 and NO-GC2 in the processing of inflammatory and neuropathic pain. The expression of NO-GC isoforms in dorsal root ganglia and the spinal cord is restricted to specific neuronal and non-neuronal cell populations. It remains to be determined which targets mediate the pain-relevant effects of NO-GC1 and NO-GC2.

